# Supplementary material for: ProteinShader: illustrative rendering of macromolecules
Source: BMC Struct Biol. 2009 Mar 30;9:19. doi: 10.1186/1472-6807-9-19 (PMC2672931; doi:10.1186/1472-6807-9-19)
Supplement: Additional file 1 — ProteinShader program without source code. This compressed file contains the complete ProteinShader program including associated libraries, but no source code. A README.txt file gives an overview of the ProteinShader distribution, and the index.html file in the help subdirectory has directions on getting started with the program as well as a set of tutorials. [file 1472-6807-9-19-S1.zip › ProteinShader-beta-0_9_4-binary/help/api/org/proteinshader/graphics/textures/NamePair.html]

NamePair (ProteinShader API)


|  |  |  |
| --- | --- | --- |
| **Overview** \| **Package** \| **Class** \| **Use** \| **Tree** \| **Deprecated** \| **Index** \| **Help** | |  |
| **PREV CLASS**   **NEXT CLASS** | **FRAMES**    **NO FRAMES**     **All Classes** |  |
| SUMMARY: NESTED \| FIELD \| CONSTR \| METHOD | DETAIL: FIELD \| CONSTR \| METHOD |  |


---


## org.proteinshader.graphics.textures Class NamePair

```
java.lang.Object
  org.proteinshader.graphics.textures.NamePair
```

---

``` public class NamePair extends Object ```

Stores a menu name and a file name for a texture.

---

**Constructor Summary**

`NamePair()`
  
          Constructs a NamePair object.

`NamePair(String menuName,
String filename)`
  
          Constructs a NamePair object.


| **Method Summary** | |
| --- | --- |
| `String` | `getFilename()`             Returns the name of the file the texture is to be read from. |
| `String` | `getMenuName()`             Returns a name suitable for use in a menu. |
| `void` | `setFilename(String filename)`             Sets the name of the file that the texture is to be read from. |
| `void` | `setMenuName(String menuName)`             Sets a menu name for the texture. |
| `String` | `toString()`             Returns the menu name and filename pair. |

**Methods inherited from class java.lang.Object**

`clone, equals, finalize, getClass, hashCode, notify, notifyAll, wait, wait, wait`

**Constructor Detail**

### NamePair

```
public NamePair()
```

:   Constructs a NamePair object.
    The menu name and filename will be set to empty Strings.

---


### NamePair

```
public NamePair(String menuName,
                String filename)
```

:   Constructs a NamePair object.

    **Parameters:**: `menuName` - a name suitable for use in a GUI menu.: `filename` - the name of the file the texture is to be read from.


**Method Detail**

### getMenuName

```
public String getMenuName()
```

:   Returns a name suitable for use in a menu.

    :   **Returns:**: A name to be used in a menu.

---


### setMenuName

```
public void setMenuName(String menuName)
```

:   Sets a menu name for the texture.

    :   **Parameters:**: `menuName` - a name suitable for use in a menu.

---


### getFilename

```
public String getFilename()
```

:   Returns the name of the file the texture is to be read from.

    :   **Returns:**: The name of the texture file.

---


### setFilename

```
public void setFilename(String filename)
```

:   Sets the name of the file that the texture is to be read from.

    :   **Parameters:**: `filename` - the name of the texture file.

---


### toString

```
public String toString()
```

:   Returns the menu name and filename pair.

    :   **Overrides:**: `toString` in class `Object`
    :   **Returns:**: The menu name and filename.


---


|  |  |  |
| --- | --- | --- |
| **Overview** \| **Package** \| **Class** \| **Use** \| **Tree** \| **Deprecated** \| **Index** \| **Help** | |  |
| **PREV CLASS**   **NEXT CLASS** | **FRAMES**    **NO FRAMES**     **All Classes** |  |
| SUMMARY: NESTED \| FIELD \| CONSTR \| METHOD | DETAIL: FIELD \| CONSTR \| METHOD |  |


---

# *Copyright © 2007-2008*
